# Detecting Vertical Root Fractures Using Modified Methylene Blue Dye: A Preliminary In Vitro Study

**DOI:** 10.3390/healthcare11040504

**Published:** 2023-02-09

**Authors:** Hadi Mohammed Alamri, Abdullah Altamimi, Mubashir Baig Mirza, Mazen A. Aldosimani, Hani Ghabbani, Fahd Aljarbou

**Affiliations:** 1 Conservative Dental Science Department, College of Dentistry, Prince Sattam bin Abdulaziz University, Alkharj 11942, Saudi Arabia; 2 Ministry of Health, Riyadh 11452, Saudi Arabia; 3 Oral Medicine and Diagnostic Sciences Department, College of Dentistry, King Saud University, Riyadh 11545, Saudi Arabia; 4 Department of Restorative Dental Sciences, Taibah University, Medina 42353, Saudi Arabia; 5 Restorative Dental Science Department, College of Dentistry, King Saud University, Riyadh 11545, Saudi Arabia

**Keywords:** CBCT, diagnosis, endodontics, radiology, vertical root fracture

## Abstract

Diagnostically, vertical root fractures (VRFs) can be a frustrating experience for the dentist. Misdiagnosis could result in significant time and effort losses involved in erroneously intervening endodontically and/or periodontally. Certainly, diagnosing VRFs is often very difficult, and diagnoses based on speculations have led to the extraction of many salvageable teeth. This study was conducted in the radiology unit of College of Dentistry, Prince Sattam bin Abdulaziz University, between December 2021 and June 2022 to evaluate the ability to detect VRFs following the use of a novel radio-opaque dye using periapical radiographs (PARs) and cone-beam computed tomography (CBCT). After carefully inducing VRFs on extracted, single-rooted, virgin premolars (n = 26), they were assigned to control (n = 2) and experimental groups (n = 24). The fracture site of the tooth in the control group received methylene blue dye, whereas the experimental group received a novel dye. Two differently angled PARs were obtained for all the teeth, followed by a CBCT image. Three blinded investigators participated in scoring a Likert scale form with a set of questions. Inter-/intra-examiner reliability showed excellent consistency using Cronbach’s alpha test. The Z-test revealed CBCT and PAR to be equally adept at detecting VRFs, with the mean values showing no statistically significant differences. The extent of the VRFs and dye penetration were significantly better when angled radiographs and axial view CBCT were evaluated. Within the limitations of this study, the dye tested showed promising initial results as an aid in radiographically detecting VRFs. The use of such minimally invasive methods is critically needed for diagnosing and managing VRFs. However, further testing should be performed prior to its clinical use.

## 1. Introduction

Diagnostically, vertical root fractures (VRFs) can be a frustrating experience for the dentist. Misdiagnosis could result in significant losses of time and effort involved in erroneously intervening endodontically and/or periodontally, based on clinical and radiographic signs such as pain and discomfort upon biting or percussion [1], localized periodontal pockets around the fractured line [2,3,4,5], and a persistent sinus tract [6,7,8]. Moreover, these signs are usually inconsistent and highly variable [9]. Certainly, diagnosing VRFs is often very difficult, and diagnoses based on speculations have led to the extraction of many salvageable teeth. The use of tests such as the bite test, visualization under transillumination, staining, and periodontal probing have all been used to aid the diagnosis [3,4].

It was long believed that VRFs initiate near the apical part of the root and propagate coronally. However, more recently, it was understood that they can initiate as small cracks in the root at any level [5,10]. VRFs are commonly seen affecting endodontically treated teeth. Nonetheless, VRFs are also reported in teeth that have not been endodontically treated, albeit to a lesser extent [11]. In reality, a much higher percentage of cracks and root fractures may go unreported. Failure to maintain recall visits or patient failure to adhere to the recall could be cited as a possible reason for the above.

Extensive research has been directed towards the use of radiography, such as periapical radiographs (PARs) and cone-beam computed tomography (CBCT), to identify VRFs. The detection of VRFs using PARs is dependent on many factors, such as the angulation, contrast, and density of the radiograph [12]. Moreover, the sensitivity of PARs in detecting VRFs in endodontically treated teeth is usually low (24%) [13]. On the other hand, CBCT provides better quality with its 3D view and permits fracture lines to be directly visualized. It has been recognized, in numerous research studies, to have better sensitivity than PARs in detecting VRFs [14,15,16]. However, its diagnostic ability is inversely proportional to the voxel size and is also hampered in multirooted teeth and in the presence of obturation materials and intra-radicular posts [17,18].

In this in vitro study, an attempt was made to assess the ability to detect VRFs using a novel radio-opaque dye utilizing straight and angled PARs and CBCT in axial and sagittal views.

## 2. Methods

This preliminary study included 26 single-rooted, freshly extracted, virgin premolars obtained from the orthodontic clinic at College of Dentistry, Prince Sattam bin Abdulaziz University (PSAU). Two teeth were used as negative controls, and the remaining 24 teeth were used as the experimental group. Prior to initiating the study, ethical approval was attained (PSAU2020027) from the Institutional Review Board (IRB) at Prince Sattam bin Abdulaziz University. The selected teeth were inspected under an operating microscope (Zumax, Suzhou, China) for signs of fractures, cracks, or severe curvatures, and such teeth were excluded. To disinfect the teeth, they were immersed in 2.5% sodium hypochlorite (Sun Chemical, Riyadh, Saudi Arabia) overnight. The roots were covered with pink wax (Dentsply Sirona, Charlotte, NC, USA) and mounted in a mixture of gypsum and wood powder to closely mimic the density of the jaw bone seen in radiographs.

To induce the fracture lines, the teeth were decoronated; a brass pin was placed in the canal, and using an Instron machine (Zwick/Roell, GmbH & Co, KG, Dettingen unter Teck, Germany), force was slowly applied until a visible/audible crack was achieved. The blocks were then placed on previously customed alginate seats and mounted on a radiograph ring to ensure reproducibility of the radiograph angle.

Methylene blue dye (Rupal Colorchem Industries, Ahmedabad, India) was used for control teeth. Fillers were added to methylene blue dye to prepare a novel dye, which was used for teeth in the experimental group. The powdered fillers within it were unified and ground using a planetary ball mill (Fritsch Pulverisette 7; Fritsch GmbH, Idar-Oberstein, Germany) for one hour at 400 rpm using zirconia balls. The average particle sizes before and after milling were measured using Zetasizer (Malvern Panalytical Ltd., Malvern, UK).

**Figure 1:**
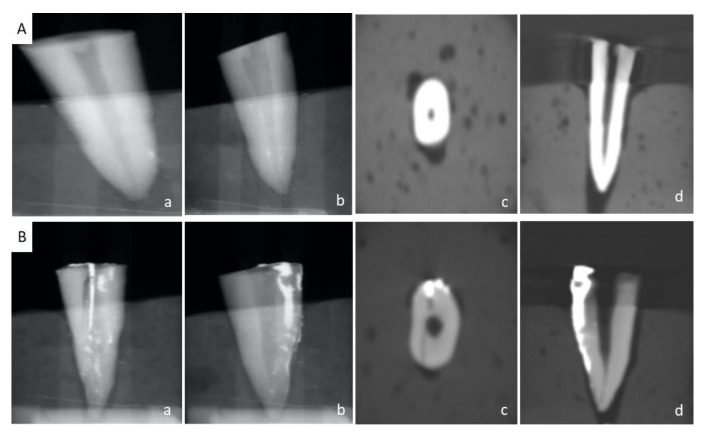
Detection of vertical root fracture in control and experimental groups. (**A**) Control group ((**a**), straight PAR; (**b**), angled PAR; (**c**), axial CBCT; (**d**), sagittal CBCT). (**B**) Experimental group ((**a**), straight PAR; (**b**), angled PAR; (**c**), axial CBCT; (**d**), sagittal CBCT).

The dyes were then placed around the circumference of the roots using a micro brush. Gentle air was blown using a three-way air syringe with the intention to force it in the crack/fracture lines. The blocks were placed on alginate seats and radiographed in straight view and tube-shift view using a Carestream CS5200 (Carestream Dent LLC, Atlanta, GA, USA) digital sensor. The CBCT images of samples were attained using a Carestream CS9300 (Carestream Dent LLC, Atlanta, GA, USA) machine with voxel sizes of 180–300 mm. The digital radiographs and CBCT images of all the 26 teeth (Figure 1) were analyzed and evaluated on a five-point Likert scale, as seen in Table 1, by three blinded dentists: two experienced endodontists and a maxillo-facial radiologist. The constructive validation of the questionnaire was performed by independent members of the IRB after going through the results of an initial pilot study performed by the authors. Based on the recommendations of the expert committee, the questionnaire was modified to evaluate the dye penetration apically along the length of the root as well as laterally towards the pulp in thirds.

### Statistical Analysis

An independent samples Z-test was used to find statistically significant differences within each method of investigation. Statistical analyses were performed using SPSS software, version 20.0 (SPSS, Inc., Chicago, IL, USA). *p*-values equal to or less than 0.05 were considered statistically significant.

## 3. Results

Cronbach’s alpha test showed excellent reliability of more than 0.98. In Table 2, comparing the images of digital radiography with questions related to the apical penetration of the dye, there was agreement among reviewers about its visibility until the middle third in both straight PARs and angled PARs. However, the responses tended to be more neutral when evaluating visibility in the apical third. For the questions related to the lateral penetration of the dye towards the root canal in each of the three thirds, the responses varied from neutral, regarding the coronal third, to disagreement on its visibility in the apical third. However, regarding the middle third, the responses were mixed between neutral and disagreement.

In Table 3, comparing the different views of CBCT for evaluating the apical penetration of dye, there was strong agreement about visibility in the coronal third in both axial and sagittal views. In axial view, the reviewers strongly agreed on its visibility in the middle third and apical third. Their responses in sagittal view varied from strongly agree to neutral with respect to the middle third and from agree to neutral with respect to the apical third. When evaluating the lateral spread of the dye towards the root canal using CBCT, with respect to the coronal third, the responses varied between strongly agree and neutral in axial view, and in sagittal view, they varied between agreement on its visibility and neutral. Regarding the axial view, the responses were in agreement with respect to the middle third, and they varied between agreement and neutral with respect to apical third, whereas in sagittal view, their responses varied from neutral to disagreement on dye visibility.

The mean values of all the samples based on the reviewers’ responses were attained, and the z-score for each of the seven questions was calculated for both PARs and CBCT images, as shown in Table 4 and Table 5, respectively. In the PAR group, when comparing dye visibility in axial PARs and sagittal PARs, significant difference was seen in all the responses, except when the visibility of the dye in the coronal third was assessed.

A significant difference among the responses was seen for all the questions, except for the general visibility of the dye and its visibility in the coronal third, on samples evaluated using axial CBCT and sagittal CBCT.

## 4. Discussion

VRFs have long been associated with endodontically treated teeth, with reported prevalence rates of 3.69–25% [11]. The main concern with such cases is that they clinically present with vague signs and symptoms, which makes management more difficult and leads to a high probability of misdiagnosing the case [3]. Some of the signs seen include the presence of V-shaped osseous defects [19], which usually leads to isolated deep pockets, J-shaped lesions seen radiographically [20], and/or a persistent sinus tract [21].

Symptoms can also vary in patients with VRFs, ranging from patients being completely asymptomatic to complaining of discomfort and pain in relation to the affected tooth [15]. In such cases, the diagnosis is usually achieved by visualizing the crack or fracture line under a dental operating microscope, either by disassembling the restoration and removal of the root-canal fillings, or by using dyes or transillumination [5]. If disassembling is not feasible, an exploratory surgery is usually performed [22]. More recently, with the availability of advanced radiographic imaging techniques and CBCT, VRFs can be more frequently determined depending on the degree of fracture [23].

Root-canal-treated teeth are more susceptible to VRFs due to the loss of tooth structure during access opening [7,24], the use of greater taper engine-driven instruments for shaping, the use of posts [4,6,15,25,26,27], and the technique used for obturation [15,27]. The choice of irrigant and the prolonged use of intra-canal medicaments such as calcium hydroxide have also been reported to affect the physical properties of dentin and render the tooth more susceptible to fracture [28,29,30]. On the other hand, in non-endodontically treated teeth, microstructural changes and increased brittleness due to the age of the patient [31,32] and the presence of para-functional habits could trigger VRFs [33,34].

Dyes have been previously used for the staining of cracks and fracture lines during microsurgical retreatment [35]. For directly visualizing the cracks along the root surface, the reflection of flaps is often required [17]. In the present study, the enhanced dye penetrated the fracture lines created and was visible in both PARs and CBCT images with a high correlation. This finding is promising, as it may aid in diagnosing possible VRFs more effectively and could help manage such cases in a more efficient way, both clinically and economically, than the currently used methods [36].

Non-invasive VRF detection has historically relied upon the appearance of clinical and radiographic signs and symptoms, which oftentimes appear in an advanced stage [1]. Conventional PARs have been less successful in detecting VRFs (35.7%), as the radiolucency is not always clear unless displacement of root fragments has occurred [37]. However, the use of differently angled PARs may be helpful [11]. More recently, comparing CBCT and intra-oral radiography, Mizuhashi et al. reported CBCT to be significantly superior in detecting VRFs [38]. Similar results were reported in other studies, also indicating that its diagnostic ability was not influenced by the presence of posts or gutta-percha [39,40]. In contrast, Dias et al. reported the use of CBCT to be non-diagnostic in endodontically treated teeth due to the loss of specificity. Currently, justification for employing CBCT to detect VRFs is insufficient [41].

In addition to the use of dye in the present study, a comparison was also performed between different angles of digital PARs to evaluate their efficiency in detecting VRFs. Similarly, VRF detection based on CBCT in different views was also compared. Differences in mean values indicate that angled PARs were better in diagnosing VRFs than straight PARs, and axial view CBCT images were better than images evaluated in sagittal view when detecting the fracture extent both apically and laterally. The early results obtained using this novel dye with the PAR technique, as they are comparable to those obtained with CBCT, could help in easing the diagnosis of VRFs, especially when CBCT is unavailable.

In spite of the perceived advantages of the study, it has some limitations: 1. As this is a preliminary study, the sample size was inestimable due to lack of published research on the tested material and the method used to assess it. 2. The biocompatibility of the dye is a critical issue to investigate, as the technique described requires blowing the dye in the sulcus, where it comes into direct contact with the periodontal tissues. Moreover, other physical properties of the dye need to be further investigated. 3. Caution is required when interpreting the results of in vitro studies because of higher chances of wider separations of fracture margins than what is encountered clinically, especially in the initial stages.

## 5. Conclusions

In view of the limitations of this study, the novel dye tested shows promising initial results as an aid in detecting VRFs in vitro. The novelty of this study lies in the radio-opaque nature of the dye, which could practically ease the interpretation of VRFs radiographically, given the difficulty experienced in their early diagnosis with current means. However, further histological testing should be performed before it is considered for clinical use.

## Figures and Tables

**Table 1:** Questionnaire.

Periapical Radiographic Evaluation	Cone-Beam Computed Tomography
Likert Scale: 1 Strongly Agree 2 Agree 3 Neutral 4 Disagree 5 Strongly Disagree	
Is the dye visible on the radiograph?	Is the dye visible on the CBCT?
Do you see the dye in the coronal third?	Do you see the dye in the coronal third?
Do you see the dye in the middle third?	Do you see the dye in the middle third of the root?
Do you see the dye in the apical third?	Do you see the dye in the apical third of the tooth?
Do you see the dye penetrating the fracture line through the dentin half way towards the root canal in the coronal third?	Do you see the dye penetrating the fracture line through the dentin half way towards the root canal in the coronal third?
Do you see the dye penetrating the fracture line through the dentin half way towards the root canal in the middle third?	Do you see the dye penetrating the fracture line through the dentin half way towards the root canal in the middle third?
Do you see the dye penetrating the fracture line through the dentin half way towards the root canal in the apical third?	Do you see the dye penetrating the fracture line through the dentin half way towards the root canal in the apical third?

**Table 2:** Percentages of means of responses based on PARs for experimental group (n = 24).

Questionnaire	Likert Scale	PARs	
		Straight View	Angled View
Is the dye visible on the radiograph?	Strongly Agree	3 (100.0%)	3 (100.0%)
Is the dye visible in the Coronal third?	Strongly Agree	3 (100.0%)	3 (100.0%)
Is the dye visible in the Middle third?	Agree	3 (100.0%)	3 (100.0%)
Is the dye visible in the Apical third?	Neutral	3 (100.0%)	3 (100.0%)
Does the dye penetrate the fracture line half way towards the root canal in the coronal third?	Neutral	3 (100.0%)	3 (100.0%)
Does the dye penetrate fracture line half way towards the root canal in the middle third?	Neutral	0 (0.0%)	2 (66.7%)
	Disagree	3 (100.0%)	1 (33.3%)
Does the dye penetrate fracture line half way towards the root canal in the apical third?	Disagree	3 (100.0%)	3 (100.0%)

Note: 3, means of scores for 24 teeth in experimental group given by three reviewers. 2, means of scores for 24 teeth in experimental group given by three reviewers. 1, means of scores for 24 teeth in experimental group given by three reviewers.

**Table 3:** Percentages of means of responses based on CBCT for experimental group (n = 24).

Questionnaire	Likert Scale	CBCT	
		Axial View	Sagittal View
Is the dye visible on the CBCT	Strongly Agree	3 (100.0%)	3 (100.0%)
Is the dye visible in the Coronal third?	Strongly Agree	3 (100.0%)	3 (100.0%)
Is the dye visible in the Middle third?	Strongly Agree	3 (100.0%)	1 (33.3%)
	Agree	0 (0.0%)	1 (33.3%)
	Neutral	0 (0.0%)	1 (33.3%)
Is the dye visible in the Apical third?	Agree	3 (100.0%)	1 (33.3%)
	Neutral	0 (0.0%)	2 (66.7%)
Does the dye penetrate the fracture line half way towards the root canal in the coronal third?	Strongly Agree	3 (100.0%)	0 (0.0%)
	Agree	0 (0.0%)	2 (66.7%)
	Neutral	0 (0.0%)	1 (33.3%)
Does the dye penetrate fracture line half way towards the root canal in the middle third?	Agree	3 (100.0%)	0 (0.0%)
	Neutral	0 (0.0%)	2 (66.7%)
	Disagree	0 (0.0%)	1 (33.3%)
Does the dye penetrate fracture line half way towards the root canal in the apical third?	Strongly Agree	1 (33.3%)	0 (0.0%)
	Neutral	2 (66.7%)	1 (33.3%)
	Disagree	0 (0.0%)	2 (66.7%)

Note: 3, means of scores for 24 teeth in experimental group given by three reviewers. 2, means of scores for 24 teeth in experimental group given by three reviewers. 1, means of scores for 24 teeth in experimental group given by three reviewers.

**Table 4:** Chi-square test based on means of Likert scale responses based on PARs for experimental group (n = 24).

Questionnaire	Group	N	Mean	Std. Deviation	Z
Is the dye visible on the radiograph?	Straight view PAR	3	25.000	0.000	2.236
	Angled view PAR	3	24.000	0.000	*p* = 0.025 *
Do you see the dye in the Coronal third?	Straight view PAR	3	24.000	0.000	0.000
	Angled view PAR	3	24.000	0.000	*p* = 1 ns
Do you see the dye in the Middle third?	Straight view PAR	3	48.667	2.887	1.993
	Angled view PAR	3	42.667	1.528	*p* = 0.046 *
Do you see the dye in the apical third?	Straight view PAR	3	66.333	1.528	1.964
	Angled view PAR	3	61.000	1.000	*p* = 0.05 *
Does the dye penetrate the fracture line half way towards the root canal in the coronal third?	Straight view PAR	3	77.667	1.528	1.964
	Angled view PAR	3	72.000	3.606	*p* = 0.05 *
Does the dye penetrate the fracture line half way towards the root canal in the middle third?	Straight view PAR	3	93.333	1.528	1.993
	Angled view PAR	3	84.333	2.309	*p* = 0.046 *
Does the dye penetrate the fracture line half way towards the root canal in the apical third?	Straight view PAR	3	99.667	0.577	2.023
	Angled view PAR	3	92.667	2.309	*p* = 0.043 *

Note: 3, means of scores for 24 teeth given by three reviewers based on PARs in experimental group. * Significance at *p*-value ≤ 0.05.

**Table 5:** Chi-square test based on means of Likert scale responses based on CBCT for experimental group (n = 24).

Questionnaire	Group	N	Mean	Std. Deviation	Z
Is the dye visible on the CBCT?	Axial view CBCT	3	24.000	0.000	0.000
	Sagittal view CBCT	3	24.000	0.000	*p* = 1 ns
Do you see the dye in the Coronal third?	Axial view CBCT	3	24.000	0.000	1.000
	Sagittal view CBCT	3	27.000	5.196	*p* = 0.317 ns
Do you see the dye in the Middle third?	Axial view CBCT	3	30.000	0.000	2.087
	Sagittal view CBCT	3	43.333	15.373	*p* = 0.037 *
Do you see the dye in the Apical third?	Axial view CBCT	3	50.000	0.000	2.087
	Sagittal view CBCT	3	60.667	3.512	*p* = 0.037 *
Does the dye penetrate the fracture line half way towards the root canal in the coronal third?	Axial view CBCT	3	28.333	0.577	2.023
	Sagittal view CBCT	3	62.333	9.238	*p* = 0.043 *
Does the dye penetrate the fracture line half way towards the root canal in the middle third?	Axial view CBCT	3	46.333	2.309	1.993
	Sagittal view CBCT	3	77.333	8.386	*p* = 0.046 *
Does the dye penetrate the fracture line half way towards the root canal in the apical third?	Axial view CBCT	3	60.000	31.177	1.993
	Sagittal view CBCT	3	87.667	8.083	*p* = 0.046 *

Note: 3, means of scores for 24 teeth given by three reviewers based on CBCT in experimental group. * Significance at *p*-value ≤ 0.05.

## Data Availability

Data are available on request.
